# An evidence based conceptual framework for the multifactorial understanding of proximal junctional kyphosis

**DOI:** 10.1016/j.bas.2024.102807

**Published:** 2024-04-20

**Authors:** Pearce B. Haldeman, Samuel R. Ward, Joseph Osorio, Bahar Shahidi

**Affiliations:** a Department of Orthopaedic Surgery, UC San Diego, La Jolla, CA, USA; b Department of Neurological Surgery, UC San Diego, La Jolla, CA, USA

**Keywords:** Proximal junctional kyphosis, Adult spinal deformity, Spine fusion, Adjacent segment disease, Conceptual framework

## Abstract

**Introduction:**

Adult spinal deformity (ASD) is a debilitating pathology that arises from a variety of etiologies. Spinal fusion surgery is the mainstay of treatment for those who do not achieve symptom relief with conservative interventions. Fusion surgery can be complicated by a secondary deformity termed proximal junctional kyphosis (PJK).

**Research question:**

This scoping review evaluates the modern body of literature analyzing risk factors for PJK development and organizes these factors according to a multifactorial framework based on mechanical, tissue or demographic components.

**Materials and methods:**

An extensive search of the literature was performed in PubMed and Embase back to the year 2010. Articles were assessed for quality. All risk factors that were evaluated and those that significantly predicted the development of PJK were compiled. The frequency that a risk factor was predictive compared to the number of times it was evaluated was calculated.

**Results:**

150 articles were reviewed. 57.3% of papers were of low quality. 76% of risk factors analyzed were focusing on the mechanical contribution to development of PJK versus only 5% were focusing on the tissue-based contribution. Risk factors that were most frequently predictive compared to how often they were analyzed were Hounsfield Units of vertebrae, UIV disc degeneration, paraspinal muscle cross sectional area and fatty infiltration, ligament augmentation, instrument characteristics, postoperative hip and lower extremity radiographic metrics, and postoperative teriparatide supplementation.

**Discussion and conclusion:**

This review finds a multifactorial framework accounting for mechanical, patient and tissue-based risk factors will improve the understanding of PJK development.

## Highlights

• Proximal junctional kyphosis (PJK) continues to occur following spine fusion.
• The most studied risk factors for PJK are mechanical in nature.
• Soft tissue structures were least often studied yet most often predictive of PJK.
• Conceptual framework for PJK includes mechanical, patient and tissue-based factors.

## 1 Introduction

Amongst the aging population, adult spinal deformity (ASD) has an estimated prevalence between 32% and 68% (Kebaish et al., 2011; Francis, 1988). ASD is a broad term comprising disorders such as spinal scoliosis, hyperkyphosis, deformity secondary to degenerative disc disease, iatrogenic, and traumatic deformity – all of which contribute to the development of chronic pain, focal neurologic deficits and poor self-image. Though more insidious, ASD is also associated with significantly higher rates of depression, anxiety and a disability burden similar to that of patients with lung cancer (Diebo et al., 2018a, 2018b), which highlights the severe morbity associated with the sydrome. Conservative management includes interventions such as physical therapy, various oral analgesic modalities, interventional local procedures, and bracing in select cases. Surgical correction, in the form of spinal fusion, however, is an often-necessary option, to improve pain and other associated disabilities after conservative measures have been exhausted and have failed.

A significant complication of spinal fusion to correct ASD is proximal junctional kyphosis (PJK), which is defined as a sagittal Cobb angle of 10–20° between the upper instrumented vertebrae (UIV) and the vertebrae two levels above (UIV+2), or 10–15° greater than the preoperative measurement (Glattes et al., 2005; Bridwell et al., 2013; O'Shaughnessy et al., 2012; Helgeson et al., 2010). The current reported incidence of PJK is between 17 and 46% (Kim et al., 2012a, 2016; Lau et al., 2014). Patients who suffer from PJK experience a similar constellation of symptoms as they did prior to surgery, including continued, or new, neurological deficits, vertebral fractures, poor self-image, debilitating pain, and overall decreased quality of life often resulting in the need for a revision surgery that is requires an extension of levels resulting in further compromise in mobility from the length of the fusion construct (Kim et al., 2016; Lau et al., 2014).

Generally, investigations aimed at surgical complications have focused on intraoperative and perioperative factors. Accordingly, there are decades of research investigating intraoperative mechanical interventions designed to decrease the risk of PJK, including which devices to use, what degree of correction to target, what approach to use, whether to use adjunctive soft tissue supports and the length and location of instrumentation (Arora et al., 2023; Sardar et al., 2021). Despite the heterogenous and multifactorial nature of ASD, both pre and postoperative factors’ (outside of the intraoperative and perioperative period) contributions to PJK risk are traditionally understudied. Postoperative metrics are generally limited to static sagittal radiographic measurements of spinal alignment and the use of postoperative bone fortifying medications. Many risk factors for PJK have been evaluated and identified in the past decade; however, the more often studied radiographic and mechanical risk factors have limited predictability (Hills et al., 2022; Johnson et al., 2023).

The role of soft tissue health – namely muscle, ligament, and disc – in the development of PJK is gaining attention (Gengyu et al., 2022). For example, studies finding muscle atrophy to be a risk factor for PJK date back to as early as 2016 (Hyun et al., 2016), Though this finding is increasingly acknowledged in recent literature (Chen et al., 2023; Zhang et al., 2022; Pinter et al., 2023), there remains a need for a scoping multifactorial framework to conceptualize this complex phenomenon (Haldeman et al., 2022). The purpose of our investigation is to provide a comprehensive review of risk factors impacting PJK across the full trajectory of patient care and to create a conceptual framework for the multifactorial contribution of preoperative, intraoperative, and post-operative risk factors for development of PJK.

## 2 Methods

A systematic review was performed according to the Preferred Reporting Items for Systematic Review and Meta-Analysis (PRISMA) guidelines (Page et al., 2021). PubMed/MEDLINE (National Library of Medicine) and Embase (Elsevier) were searched by one author (P.B.H.) in March 2022 using the following search term: "Proximal Junctional Kyphosis" OR "Proximal Junctional Failure". The results were restricted to a published year as early as 2010. A restriction was chosen so as to include the majority of only contemporary literature on this topic. A histogram of years in which this topic was published was consulted, and an inflection point was noted at the year 2010. There were no restrictions on language, data, or article type upon initial search.

**Fig. 1:**
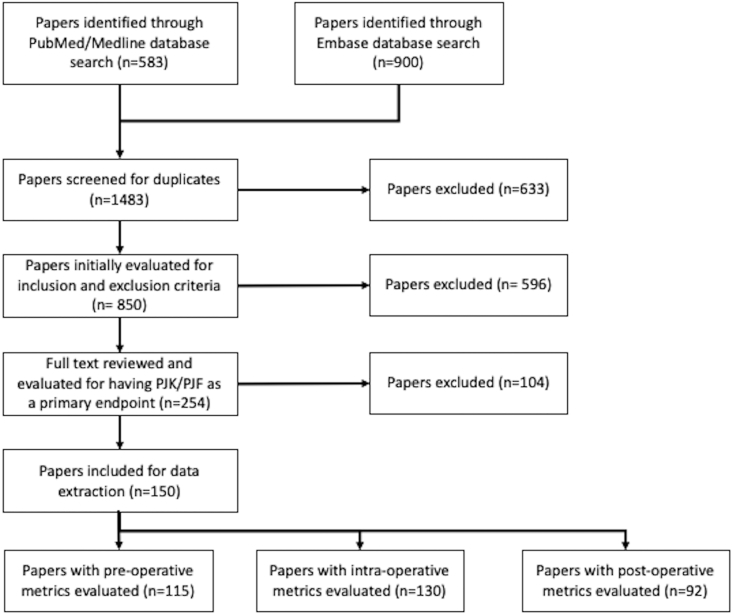
PRISMA Diagram for literature search and selection.

After completing the search, duplicates were excluded. Remaining articles were screened for the following inclusion criteria: 1) published or translated into English, 2) presenting primary data, 3) population with mean age >18 years; and exclusion criteria: 1) conference abstracts, 2) case reports, 3) *in silico* studies, 4) technical notes and 5) exclusively ankylosing spondylitis, rheumatoid arthritis or osteogenesis imperfecta populations. Finally, records were reviewed by two authors (P.B.H. and B.S.) to evaluate if PJK was included as a primary endpoint. Records were also stratified according to their evaluation of pre, intra, or postoperative risk factors for PJK development (Fig. 1).

Each record was evaluated for quality by applying the Grading of Recommendations, Assessment, Development and Evaluations (GRADE) criteria (Guyatt et al., 2011a, 2011b, 2011c, 2011d, 2011e, 2011f, 2011g, 2011h, 2013; Balshem et al., 2011; Mustafa et al., 2013) (Table 1). Discrepancies were resolved by discussion between two authors (P.B.H. and B.S.). No meta-analysis was conducted given heterogeneity in study designs and outcomes of the included studies.

**Table 1:** Certainty of conclusion evaluation using GRADE criteria for included papers.

Records	Type of Evidence	Risk of Bias	Precision	Consistency	Directness	Certainty
(Li et al., 2022; Yagi et al., 2022; Kaufmann et al., 2022; Takasawa et al., 2022; Katsuura et al., 2022; Lafage et al., 2017a, 2020; Park et al., 2020a; Kim et al., 2020a; Daniels et al., 2019; Line et al., 2020; Pennington et al., 2019; Sebaaly et al., 2018a; Nicholls et al., 2017)	2	0	1	0	0	Moderate
(Bridwell et al., 2013; O'Shaughnessy et al., 2012), (Hills et al., 2022), [48-130]	2	0	0	0	0	Low
(Kolz et al., 2022; Kim et al., 2013)	2	−1	1	0	0	Low
(Hyun et al., 2016), (Liao et al., 2022; Tsutsui et al., 2022; Lord et al., 2021; Sakuma et al., 2021; Cazzulino et al., 2021; Harris et al., 2021; Denduluri et al., 2021; Funao et al., 2021; Yao et al., 2021b; McDonnell et al., 2020; Lee et al., 2020; Hasegawa et al., 2021; Eleswarapu et al., 2022; Ha et al., 2021; Taneichi et al., 2020; Park et al., 2020c; Ha et al., 2019; Ohba et al., 2018; Matsumura et al., 2018; Zhao et al., 2018; Yasuda et al., 2017; Raman et al., 2017; Sun et al., 2017; Park et al., 2017a; Kim et al., 2017; Park et al., 2017b; Yan et al., 2017; Glassman et al., 2016; Nasto et al., 2016; Yagi et al., 2014; Lee et al., 2014; Hassanzadeh et al., 2013; Theologis et al., 2015)	2	0	−1	0	0	Very low
Vital et al. (2021)	2	−1	0	0	−1	Very low
(Ohba et al., 2022; Moon et al., 2020; Arima et al., 2018; Protopsaltis et al., 2018; Wang et al., 2016; Fujimori et al., 2014)	2	−1	0	0	0	Very low
Im et al. (2020)	2	0	0	−1	0	Very low
(Kikuchi et al., 2021; Choi et al., 2019; McClendon et al., 2016)	2	−1	−1	0	0	Very low
Yilgor et al. (2018)	2	−1	0	−1	−1	Very low
(Park et al., 2018; Zhu et al., 2018)	2	0	0	0	−1	Very low

**Table 2:** List of risk factors investigated across all reviewed papers. The frequency they were evaluated and rate at which they were found to be predictive is reported.

	Preoperative			Intraoperative			Postoperative		
Mechanical	**Variable**	**Evaluated (n)**	**Predictive risk factor**	**Variable**	**Evaluated (n)**	**Predictive risk factor**	**Variable**	**Evaluated (n)**	**Predictive risk factor**
	Thoracic Kyphosis (TK)	69	18%	Surgical Approach	54	19%	Thoracic kyphosis	62	29%
	PI-LL	57	11%	Global correction	81	27%	PI-LL	51	18%
	UIV Angle	6	0%	Thoracic correction	35	26%	Global alignment	130	15%
	Global alignment	133	20%	Lumbar correction	68	21%	UIV angle	9	33%
	Coronal deformity	19	5%	Pelvic correction	49	10%	Coronal deformity	12	0%
	Lumbar spine alignment	69	6%	Fused Levels	45	16%	Lumbar spine alignment	69	28%
	Pelvic parameters	172	10%	UIV	71	39%	Pelvic parameters	130	30%
	Hip/LE metrics	6	0%	LIV	51	29%	Hip/LE metrics	4	75%
				Instrument characteristics	45	42%			
Tissue	BMD	46	37%	Posterior Junctional Tethering/Ligament Augmentation	12	67%	Teriparatide	2	100%
	Hounsfield Units (HU) of Vertebrae	5	100%	Mersilene Tape Ligament Augmentation	2	100%	Brace	1	0%
	Teriparatide /bisphosphonate	2	0%	Cement Augmentation/Vertebroplasty	13	23%			
	Fatty infiltration	4	50%	UIV Vertebroplasty	2	0%			
	Paraspinal muscle CSA	8	75%	BMP	3	33%			
	CSA of UIV disc	1	0%						
	UIV disc degeneration	1	100%						
Demographic	Age	95	18%				Comorbidities	20	15%
	BMI	61	7%				Patient reported outcomes	25	24%
	Sex	67	6%						
	Patient reported outcomes	45	2%						
	Comorbidities	26	23%						
	Spine pathology history	20	15%						

All included records underwent a thorough full article review and data extraction. This consisted of recording all reported independent variables (predictor risks) for PJK development that were evaluated in each study and stratifying them based on if they were mechanical, tissue, or demographic in nature. If a risk factor was demonstrated to have statistical significance with respect to predicting PJK, it was captured as such. Given the volume of individual factors identified, common risks were categorized according to region or construct. For example, pelvic incidence, pelvic tilt and sacral slope were condensed into a single category entitled, “pelvic parameters”. A list of all individual parameters and their respective condensed categories is provided in Supplementary Table 1. Finally, the percentage of instances in which a risk factor was statistically significant relative to the total number of papers reporting that risk factor was included in the literature was calculated.

## 3 Results

### 3.1 Study selection

**Fig. 2:**
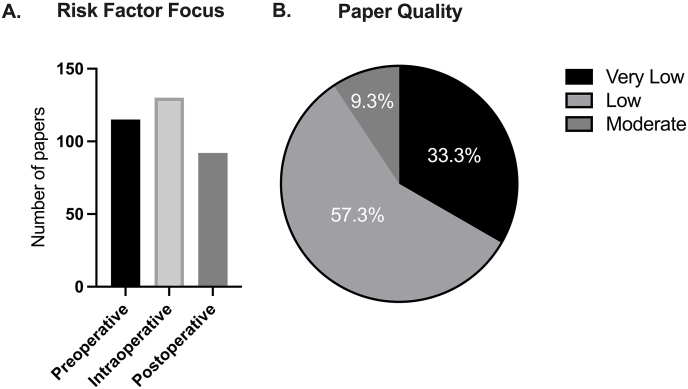
Number of articles according to procedural chronology (a) and proportion of papers according to quality (b).

A total of 1483 studies were identified when searching PubMed and Embase databases. 850 studies remained after duplicates were removed. 254 were included for full text review and 150 underwent data extraction (Fig. 1). Of those records, 115 included assessment of preoperative risk factors, 130 included intraoperative and 92 included postoperative factors (Fig. 2a).

### 3.2 Quality assessment

When the GRADE criteria were applied to all 150 studies that underwent data extraction, 14 were deemed to be of moderate quality (MQ), 86 were low quality (LQ), and 50 were very low quality (VLQ) (Table 1, Fig. 2b). 14 studies received 1 point increase in their valuation due to their increased precision and were assigned MQ. 84 studies were assigned LQ based on their study design and did not have a decrease or increase in their valuation. 2 studies had a 1-point increase in valuation for precision but also had a decrease in valuation due to risk of bias being present, thus remained LQ. 36 VLQ studies received a decrease in valuation for imprecision. 1 VLQ study received a decrease for risk of bias and indirectness. 6 VLQ studies received a decrease for risk of bias. 1 VLQ study received a decrease for inconsistency. 3 VLQ studies received a decrease for risk of bias and imprecision. 1 VLQ study received a decrease for risk of bias, inconsistency, and indirectness. 2 VLQ studies received a decrease for indirectness (Table 1).

### 3.3 Publication focus and significance rate

**Fig. 3:**
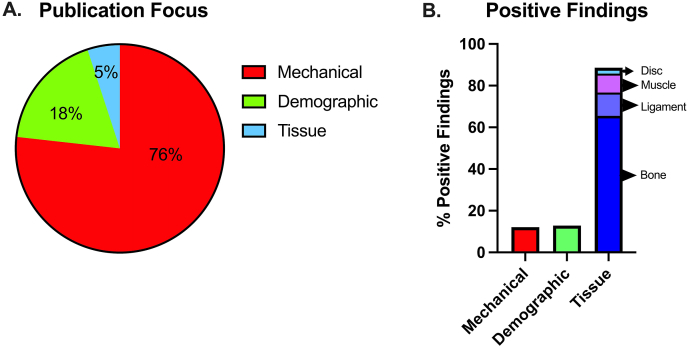
Distribution of publication focus across mechanical, tissue, and demographic-based risk factors for PJK (a), and proportion of risk factors that are significantly predictive within each category (b).

Across all publications, the majority of risk factors studied were focused on mechanical features (76%), whereas tissue-based features were the least studied (5%) (Fig. 3a). Of the studies including mechanical risk factors, 20% of the variables were found to be significant, whereas 12% of the demographic risk factors were significant, and 46% of the tissue-based risk factors were found to be significant. When tissue-based risk variables were further broken down to determine the tissue of interest, muscle tissue impairments were significant 89% of the time, bony features were 38% of the time, disc features were significant 50% of the time, and ligament features were significant 71% of the time (Fig. 3b).

### 3.4 Risk factors driving PJK (Table 2)

#### 3.4.1 Mechanical risk factors

Preoperative mechanical alignment factors were evaluated most often across all categories, and amongst them, pelvic alignment parameters were most frequently studied (172 papers), though, were only found to be statistically significant in 10% of papers. Conversely, global alignment was evaluated the second most often (133 papers) but was found to be most often significant (20%), followed by thoracic kyphosis (18%) amongst preoperative mechanical factors. Upper instrumented vertebrae (UIV) angle and Hip/Lower Extremity metrics were not often studied and were never found to be significant predictors. Amongst mechanical considerations, intraoperative factors that were most predictive of PJK development were instrument characteristics (42%) and UIV location (38%). The positivity rates for degree of global, thoracic or lumbar correction were similar, ranging from 21 to 27%, with pelvic correction demonstrating the lowest positivity rate at 10%. Postoperatively, hip and lower extremity (LE) metrics were the most positive risk factor (75)%, although this rate was based on a very low paper number (4 papers). Global alignment and pelvic parameters were studied most prevalently (130 papers each) but had relatively low rates of positivity (15% and 30% respectively). Interestingly, coronal deformity was negative for all included studies.

#### 3.4.2 Tissue-based risk factors

Bone mineral density (BMD) was the most frequently studied preoperative tissue-based risk factor, accounting for over 65% of the tissue-based papers. Vertebral Hounsfeld Units (HU's) and UIV disc degeneration were always found to be significant predictors of risk though were very seldom studied. Paraspinal muscle cross sectional area (CSA) was the second most often studied preoperative tissue-based factor, representing approximately 13% of the tissue-based studies, and was significant in 75% of papers. The most commonly studied intraoperative tissue-based risk factor was cement augmentation/vertebroplasty, representing 41% of papers. This was followed by studies on ligament augmentation with Mersilene tape or posterior junctional tethering, demonstrating 100% and 67% predictivity respectively. Postoperative supplementation with teriparatide was only studied twice in the literature but was found to be a significant predictor of decreased risk in both studies, whereas there was no positive evidence for postoperative bracing.

#### 3.4.3 Demographic risk factors

Amongst preoperative demographic factors, associated comorbidities and age were most and second most often significant, respectively, whereas preoperative patient reported outcomes were the least often significant. Conversely, amongst postoperative demographic factors, patient reported outcomes were most often significant. Preoperative BMI and sex were not often found to be significant.

## 4 Discussion

**Fig. 4:**
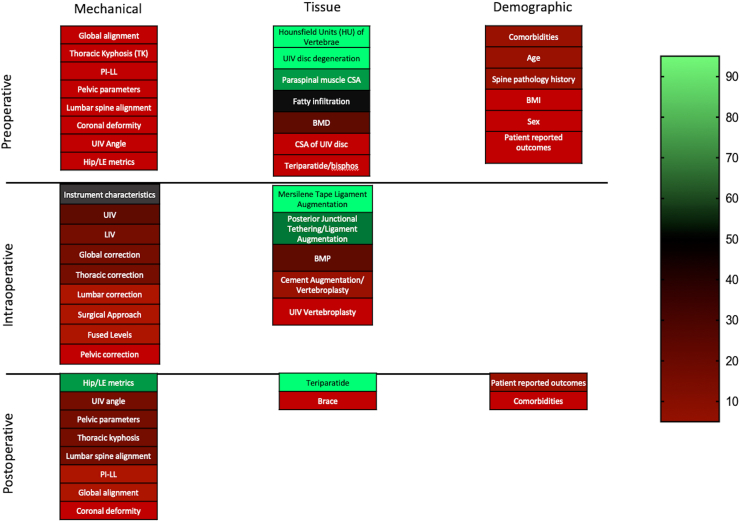
Heatmap of significant evidence for each risk factor category. Green color indicates large proportion of evidence is predictive, red indicates small proportion of evidence is predictive. (For interpretation of the references to color in this figure legend, the reader is referred to the Web version of this article.)

This systematic review explored the body of literature analyzing risk factors for the development of PJK since the year 2010. Overall, we found the majority of literature in this area is of low quality and is driven by retrospective investigations of mechanically focused intraoperative risk factors. Interestingly, despite the preponderance of literature on mechanical risk factors, our findings suggest that tissue-specific factors related to muscle, bone and ligament health, are the most consistently significant predictors of PJK development. Specifically, our findings demonstrate that higher preoperative bone density as measured by Hounsfield Units, greater preoperative paraspinal muscle size, and postoperative use of teriparatide are consistently protective against PJK development. Of mechanical and alignment-based risk factors, only postoperative hip and lower extremity alignment metrics demonstrated consistent significance, although this observation may be influenced by publication bias due to its low prevalence (only 4 papers). Similarly, no demographic characteristics demonstrated significance a majority of the time. A visual representation of the consistency of a given risk factor for significance can be found in Fig. 4.

Only in the past 2 years has the role of tissue health in development of PJK become more recognized in the context of summary literature reviews (Gengyu et al., 2022; Chen et al., 2023; Dubousset et al., 2023; Rahmani et al., 2022; Han et al., 2022). However, many reviews still focus on intraoperative mechanical or alignment-based metrics (Sardar et al., 2021; Shlobin et al., 2022). Historically, tissue health, particularly soft tissue health (e.g. muscle and ligament) seems to be relatively overlooked in surgical practice. This may be secondary to the perception that, in the hands of the surgeon, the most modifiable and actionable component of ASD treatment occurs with surgical approach and correction of malalignment. Although technically this may be the area where there is most room for surgical change, it does not preclude the idea that a key part of managing a surgical candidate occurs outside of the intraoperative window (i.e. preoperative testing and planning). As such, the surgeon may provide essential preoperative education and recommend interdisciplinary management across the full trajectory of care to optimize surgical outcomes.

For example, current guidelines suggest improving bone health prior to surgery to reduce poor outcomes is warranted (Management of Osteoporosis in Postmenopausal Women: The Position Statement of The North American Menopause Society'' Editorial and P, 2021), however, use of pharmacologic adjuvants or physical activity recommendations by surgeons in the presence of known osteoporosis is still low (Jain et al., 2022). Similarly, although many patients undergo some form of conservative management prior to surgical authorization, this management is often designed for pain control (e.g. injections, pharmacological pain management, education, or bracing), and the concept of “prehabilitation” with a focus on improving health of the paraspinal muscles around the boundaries of the planned surgical construct is not typical (Eubanks et al., 2023), despite evidence suggesting its efficacy. Generally, reducing PJK risk may require a more intentional peri-operative management strategy that incorporates specific non-surgical management to optimize tissue health pre- and postoperatively - as directed by the surgeon in accordance with the planned surgical procedure.

**Fig. 5:**
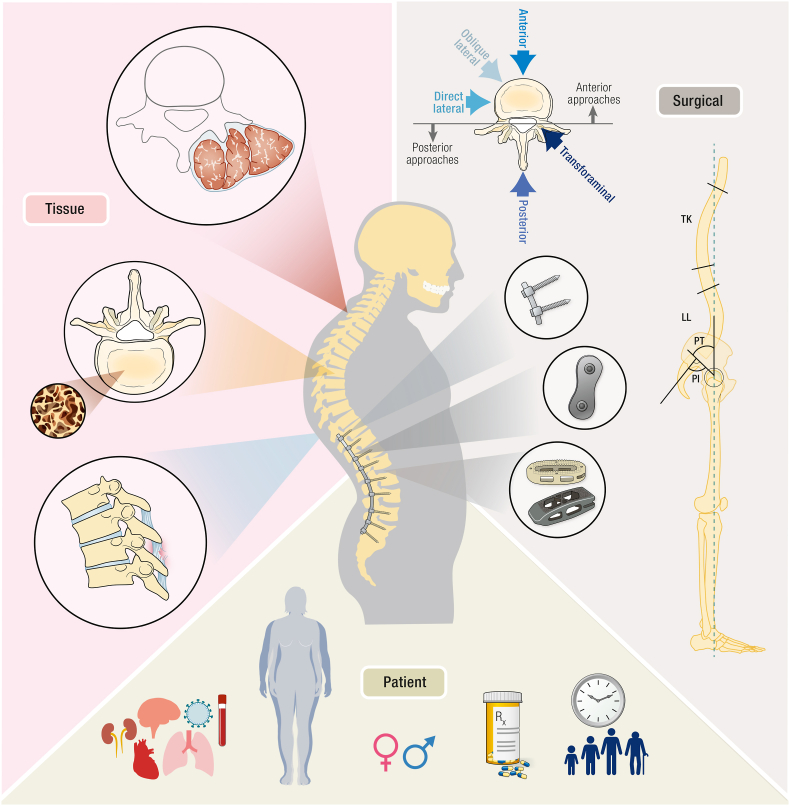
Conceptual framework for the understanding of proximal junctional kyphosis.

This review of the literature suggests that a multidimensional approach may improve our understanding of the factors leading to PJK development. Considering that postoperative loss of sagittal balance occurs secondary to a variety of interacting etiologies, an enhanced knowledge of the relationship between spinal biomechanics and soft tissue physiology will help usher spine surgery into the era of personalized medicine. This new, broad evidence-based framework (Fig. 5) serves to integrate under-recognized and emerging risks into the current body of evolving and well-recognized operative and biomechanical risk factors for the development of PJK.

These data and recommendations are not without limitations. As illustrated above, the literature is predominantly of low quality and retrospective in design. Considering high quality evidence is mostly reserved for prospective studies, and that risk factor studies are, generally, epidemiological in nature, this finding is not unexpected. This does, however, provide a degree of uncertainty, which one must have when interpreting conclusions. In light of the discrepancy between the volume of studies where mechanical factors are analyzed, and their relative significance, prospective studies taking a multifaceted and multiphase view of risk are needed. Additionally, it should also be noted that of the 150 papers that underwent full text review in our analysis, it was found that single databases, such as the International Spine Study Group (ISSG) database, were often used by multiple different authors, possibly leading to a bias in the factors that were analyzed.

## Funding

Not applicable.

## Availability of data and material

Not applicable.

## Code availability

Not applicable.

## Ethics approval

Not applicable.

## Consent to participate

Not applicable.

## Consent for publication

Not applicable.

## Declaration of competing interest

The authors declare the following financial interests/personal relationships which may be considered as potential competing interests: Samuel R. Ward reports a relationship with San Diego Spine Foundation that includes: board membership. Samuel R. Ward reports a relationship with 10.13039/100007064NuVasive Inc that includes: funding grants. If there are other authors, they declare that they have no known competing financial interests or personal relationships that could have appeared to influence the work reported in this paper.
